# Targeted BDNF upregulation via upstream open reading frame disruption

**DOI:** 10.1016/j.ymthe.2025.12.024

**Published:** 2025-12-11

**Authors:** Ning Feng, Thomas Goedert, Nenad Svrzikapa, Dongnan Yan, Hans J. Friedrichsen, Britt Hanson, Alicia Ljungdahl, Ruxandra Dafinca, Kevin Talbot, Stephan J. Sanders, Dhanu Gupta, Mathew J.A. Wood, Thomas C. Roberts

**Affiliations:** 1 Institute of Developmental and Regenerative Medicine, University of Oxford, IMS-Tetsuya Nakamura Building, Old Road Campus, Roosevelt Dr, Headington, Oxford OX3 7TY, UK; 2 Department of Paediatrics, University of Oxford, Roosevelt Dr, Headington, Oxford OX3 7TY, UK; 3 Orfonyx Bio Ltd, BioEscalator, University of Oxford, Innovation Building, Rm 10.15, Roosevelt Drive, Oxford OX3 7FZ, UK; 4 Nuffield Department of Women’s and Reproductive Health, University of Oxford, John Radcliffe Hospital, Oxford OX3 9DU, UK; 5 Department of Physiology, Anatomy and Genetics, University of Oxford, South Parks Road, Oxford OX1 3QX, UK; 6 Oxford Motor Neuron Disease Centre, Nuffield Department of Clinical Neurosciences, University of Oxford, John Radcliffe Hospital, Oxford OX3 9DU, UK; 7 Department of Psychiatry and Behavioral Sciences, UCSF Weill Institute for Neurosciences, University of California, San Francisco, San Francisco, CA 94158, USA; 8 New York Genome Center, New York, NY 10013, USA; 9 MDUK Oxford Neuromuscular Centre, Oxford OX3 7TY, UK

**Keywords:** uORF, upstream open reading frame, BDNF, brain-derived neurotrophic factor, base editing

## Abstract

To understand the relative contributions of 5ʹ UTR elements to translation output, we performed a comprehensive analysis of upstream open reading frames (uORFs) in the 5ʹ UTRs of the *BDNF* (brain-derived neurotrophic factor) transcripts. Predicted uORFs were identified in 14 out of 17 *BDNF* RefSeq transcript isoforms, and we experimentally validated five of these transcripts as being uORF-repressed, suggesting that uORF elements play an important role in shaping the protein output from this locus. We explored several approaches to disrupt *BDNF* uORF function. Deletion of a 5ʹ UTR exon in *BDNF* v11 (containing eight predicted uORFs), in order to simulate an exon skipping outcome, resulted in pronounced upregulation in a reporter construct system. This effect was found to be partially uORF-dependent but was also dependent on the disruption of an RNA secondary structure element. However, this transcript variant was found to not be expressed in human brain. Conversely, direct disruption of a single uORF start codon in the widely expressed *BDNF* v4 transcript variant using an adenine base editing approach resulted in a ∼1.8-fold upregulation of endogenous BDNF protein expression in cell culture. This study characterizes uORF-mediated regulation of the *BDNF* locus and demonstrates the potential for BDNF protein upregulation via base editing-mediated uORF disruption.

## Graphical abstract

**Figure:**
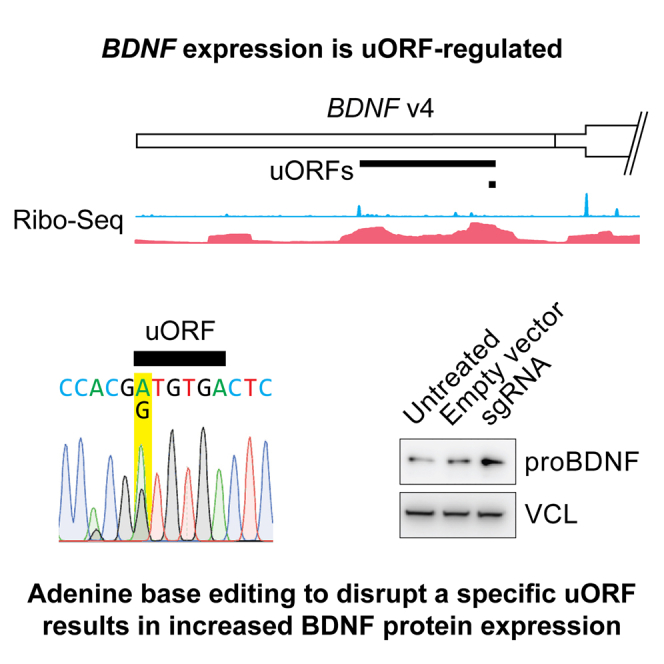

## Teaser

Roberts and colleagues report a method to upregulate expression of brain-derived neurotrophic factor (BDNF) protein by disrupting the start codon of an upstream open reading frame (uORF), which otherwise acts as a translational repressor. This approach might be more generally used for therapeutic upregulation of other specific proteins.

## Introduction

Brain-derived neurotrophic factor (BDNF) is a secreted growth factor of the neurotrophin family that plays an important role in both the development and maintenance of the nervous system. BDNF promotes neuronal cell survival and differentiation by binding to neurotrophic tyrosine kinase receptor 2 (NTRK2), also known as tropomyosin-related kinase B (TrK B).^1^^,^^2^ As such, BDNF contributes to learning and memory by increasing dendritic branching, long-term potentiation, and synaptic plasticity.^3^^,^^4^^,^^5^^,^^6^ A common valine-to-methionine substitution at BDNF residue 66 (rs6265) has been reported to result in reduced BDNF protein secretion and memory deficits,^7^ and reduced BDNF levels have been associated with a multitude of neurological and psychiatric disorders including Alzheimer’s disease,^8^^,^^9^ Parkinson’s disease,^10^^,^^11^ Huntington’s disease,^12^^,^^13^^,^^14^ Rett syndrome,^15^ and depression.^16^^,^^17^^,^^18^^,^^19^ Upregulating BDNF levels has been shown to improve symptoms in a mouse model of Rett syndrome,^20^ and reduce neuronal loss and improve pathology in various animal models of Alzheimer’s disease,^21^ Parkinson’s disease,^22^^,^^23^ Huntington’s disease,^24^ and amyotrophic lateral sclerosis (ALS).^25^ Moreover, given its neuroprotective effects, the administration of BDNF is being investigated as a possible therapeutic strategy for the treatment of acute injuries, with the aim of limiting the extent of damage following stroke,^26^^,^^27^ spinal cord injury,^28^ and traumatic brain injury.^29^

A variety of methodologies have been explored to increase BDNF levels, including delivery of recombinant protein,^30^ peptide mimetics of BDNF,^31^ gene therapy,^32^ unsilencing via targeting of a natural antisense transcript at the *BDNF* locus,^33^ and small molecule repurposing.^34^^,^^35^ Indeed, recombinant BDNF protein has been investigated in clinical trials for ALS,^36^ and a *BDNF* gene therapy phase 1 clinical trial is currently under way for the treatment of Alzheimer’s disease (NCT05040217).

Human transcripts frequently contain upstream open reading frames (uORFs), which consist of an AUG start codon located within the 5ʹ UTR followed by an in-frame stop codon. The presence of one or more uORFs is typically associated with translational repression of the downstream primary open reading frame (pORF)^37^ and, in some cases, nonsense-mediated decay.^38^ Predicted uORFs are present in more than 50% of human transcripts, suggesting that a large proportion of the human transcriptome may be held in a semi-repressed state.^39^ As such, disruption of uORF-mediated regulation provides an opportunity to relieve this repression, and thereby achieve targeted upregulation of a specific therapeutically relevant protein, such as BDNF. Regulation of rat *Bdnf* expression by untranslated regions has previously been reported.^40^ However, a thorough characterization of uORF-mediated BDNF control is currently lacking.

Here we have identified multiple uORF-regulated *BDNF* transcript isoforms, offering potential for therapeutic manipulation. uORF-mediated regulation was experimentally confirmed for five *BDNF* transcripts. *BDNF* transcript v11 (NM_001143811.2) includes a spliced 5ʹ UTR, whereby the removal of one exon was found to result in pronounced upregulation that was dependent on uORF activity and the presence of an RNA secondary structure element. Characterization of the *BDNF* transcriptional landscape in human brain tissue identified *BDNF* transcript v4 (NM_001709.5) as the optimal target transcript, which contains two uORFs. Disruption of the start codon for one of the uORFs using an adenine base editing approach resulted in up to a 2-fold increase in endogenous BDNF protein expression. This study constitutes proof-of-concept for uORF-mediated BDNF upregulation and provides a blueprint for targeted protein upregulation at other gene loci.

## Results

### Analysis of the transcriptional landscape and predicted uORFs at the *BDNF* locus

**Figure 1:**
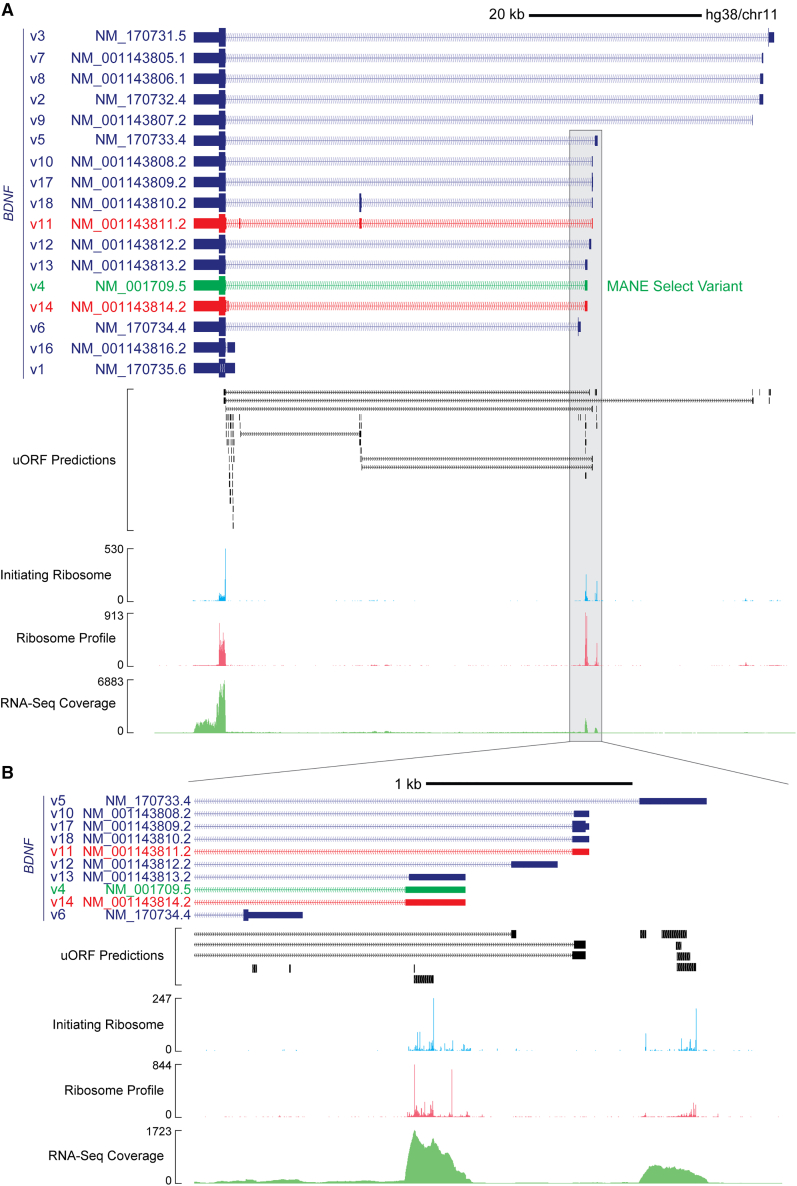
Analysis of predicted uORFs at the *BDNF* locus (A) Genome browser screenshot of the *BDNF* locus showing the 17 RefSeq transcript isoforms. The MANE Select variant is highlighted in green. Two transcripts with skippable 5ʹ UTR exons are highlighted in red (NM_001143811.2 and NM_001143814.2). Positions of predicted uORFs are indicated and the data are combined with publicly available Ribo-seq and RNA-seq data from the GWIPS-viz browser. (B) Zoomed in view of the first exons for nine transcript isoforms with Ribo-seq evidence of translation at certain uORFs.

The human *BDNF* locus was analyzed to identify predicted uORFs with publicly available ribosome profiling (Ribo-seq)/RNA-sequencing (RNA-seq) data overlaid (Figure 1). The *BDNF* gene consists of 17 RefSeq transcripts, of which 14 were predicted to contain at least one uORF (Table S1). Thirteen of these transcripts encode an identical 248-amino-acid protein, while the remaining four transcripts encode various N-terminally extended BDNF protein isoforms. The transcripts primarily differ in their transcriptional start sites and splicing of 5ʹ UTR exons.

### Multiple *BDNF* transcript isoforms are regulated by uORFs

**Figure 2:**
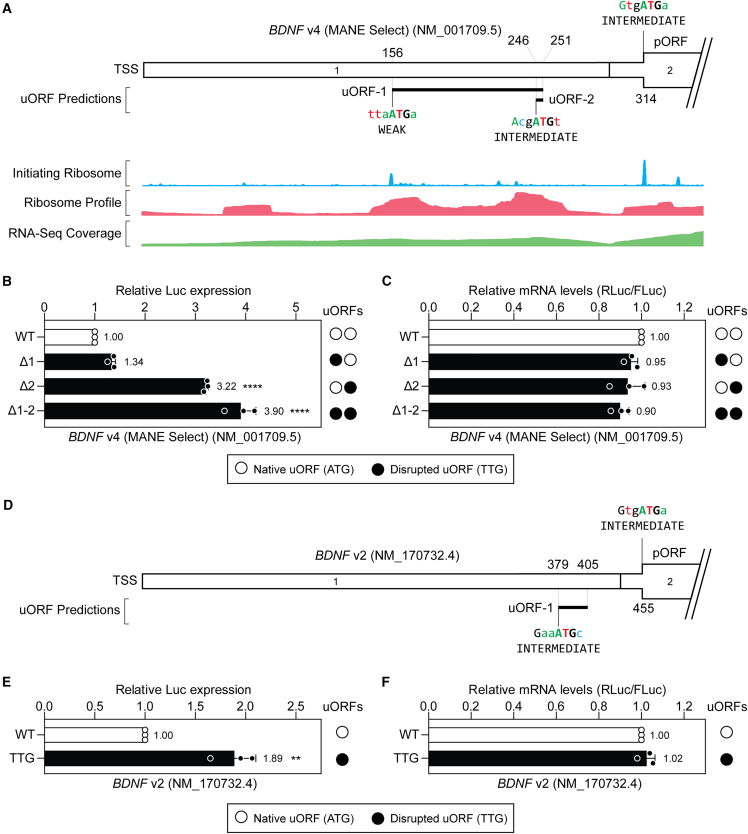
*BDNF* transcript isoforms are subject to uORF-mediated translational repression (A) Schematic of *BDNF* transcript v4 NM_001709.5 (the MANE Select variant), containing two predicted uORFs (i.e., uORF-1 and uORF-2). Aggregated Ribo-seq and RNA-seq data are overlaid providing evidence of uORF translation. (B) HEK293T cells were transfected with *BDNF* v4 5ʹ UTR dual luciferase reporter constructs as indicated and luciferase activity assayed after 24 h. uORFs were disrupted by mutation of the corresponding upstream ATG to TTG. (C) mRNA transcript levels (*Renilla* luciferase and firefly luciferase) were analyzed by RT-qPCR in separate cultures. (D) Schematic of *BDNF* transcript v2 NM_170732.4, containing one predicted uORF (uORF-1). (E) HEK293T cells were transfected with *BDNF* v2 5ʹ UTR dual luciferase reporter constructs and luciferase activity assayed after 24 h as described above. (F) mRNA transcript levels were determined in separate cultures as described above. Values are mean + SD (*n* = 3 independent experiments) and were scaled such that the mean of the WT control group was returned to a value of 1. Statistical significance was determined by one-way ANOVA with Bonferroni post hoc test and Student’s *t* test, as appropriate. ∗∗*p* < 0.01, ∗∗∗∗*p* < 0.0001.

We next sought to experimentally validate uORF functionality in various *BDNF* isoforms using a dual luciferase reporter system. The appropriate *BDNF* 5ʹ UTR was cloned upstream of a *Renilla* luciferase transgene and mutants were generated in which uORFs were disrupted by converting their respective ATG start codons to TTG, thereby ablating their activity. A separate firefly luciferase cassette served as an internal control. Initially, we focused on the MANE Select (Matched Annotation from NCBI and EMBL-EBI) transcript isoform (*BDNF* v4, NM_001709.5, ENST00000356660.9). This transcript was predicted to contain two uORFs, the second of which is a minimal uORF (i.e., consisting of a start codon followed immediately by a stop codon) (Figure 2A).

HEK293T cells were transfected with the *BDNF* v4 reporter plasmid constructs and dual luciferase activity was assessed 24 h later. Disruption of both uORFs resulted in a 3.9-fold increase in reporter expression (*p* < 0.0001) (Figure 2B). Disruption of uORF-1 (weak Kozak context) resulted in an ∼1.3-fold increase in reporter expression that did not reach statistical significance, suggesting that its contribution to this repressive effect is minimal. By contrast, disruption of uORF-2 (intermediate Kozak context) resulted in an ∼3.2-fold increase in reporter expression (*p* < 0.0001). Differences in luciferase activity between experimental groups could not be explained by altered mRNA transcript levels (Figure 2C). These findings suggest that the *BDNF* MANE Select transcript isoform is subject to a high level of uORF-mediated translational repression. This activity is mostly due to uORF-2 (a minimal uORF), although disruption of uORF-2 alone did not reach the same level of de-repression as simultaneous disruption of both uORFs, suggesting that there is an additive repressive effect of these two uORFs in series.

A second *BDNF* transcript isoform (*BDNF* v2, NM_170732.4) was analyzed in parallel. This transcript contains a single-predicted uORF in a pORF-proximal position (Figure 2D). Disruption of this uORF resulted in an ∼1.9-fold increase in reporter expression (*p* < 0.01) (Figure 2E), which could not be explained by changes in mRNA transcript levels (Figure 2F). Both *BDNF* transcript variants v2 and v4 might potentially be targeted for therapeutic BDNF protein elevation via disruption of a single uORF.

A third *BDNF* transcript isoform (*BDNF* v5, NM_170733.4) was similarly investigated. This transcript contains five predicted uORFs (Figure S1). Mutant constructs were generated in which various combinations of uORFs were disrupted as described above. Disruption of all five uORFs resulted in an ∼3.6-fold increase in reporter expression (*p* < 0.0001). However, disruption of any single uORF alone resulted in minimal (or negligible) de-repression that was not significantly different from the wild-type control (Figure S1). These data suggest that there is redundancy in function between uORFs arranged in series within the same 5ʹ UTR, such that this transcript is not a suitable target for a single uORF-targeted disruption approach.

**Figure 3:**
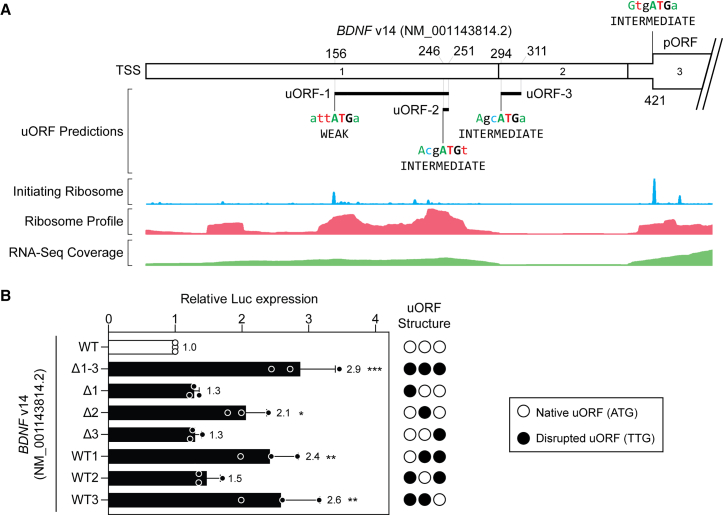
*BDNF* transcript v14 is subjected to uORF-mediated repression (A) Schematic of *BDNF* transcript v14 NM_001143814.2, containing three predicted uORFs. Aggregated Ribo-seq and RNA-seq data are overlaid providing evidence of uORF translation. (B) HEK293T cells were transfected with *BDNF* v14 5ʹ UTR dual luciferase reporter constructs as indicated and luciferase activity assayed after 24 h. uORFs were disrupted by mutation of the corresponding upstream ATG to TTG. Values are mean ± SD (*n* = 3 independent experiments) and were scaled such that the mean of the WT control group was returned to a value of 1. Statistical significance was determined by one-way ANOVA with Bonferroni post hoc test, ∗*p* < 0.05, ∗∗*p* < 0.01, ∗∗∗*p* < 0.001.

A fourth *BDNF* transcript isoform (*BDNF* v14, NM_001143814.2) was also studied. This transcript contains the same first exon as the MANE Select isoform (*BDNF* v4), containing two uORFs, described above, but also contains an alternatively spliced second exon containing a third uORF (Figure 3A). Disruption of all three uORFs resulted in an ∼2.9-fold increase in reporter expression (*p* < 0.001), whereas disruption of uORF-2 resulted in an ∼2.1-fold increase (*p* < 0.05) (Figure 3B). Conversely, a construct containing only uORF-2 was found to be highly repressive, leading to reporter levels that were not significantly different from the wild-type (WT) control construct. These data suggest that uORF-2 is responsible for the majority of translation repression activity. Notably, uORF-2 is the same minimal uORF (i.e., a start codon immediately followed by a stop codon) that was found to be repressive in the context of the MANE Select variant (*BDNF* v4) described above (Figure 2B).

### Potential for BDNF upregulation via upstream exon exclusion

Splice modulating oligonucleotides targeting the 5ʹ UTR have the potential to exclude untranslated exons containing uORFs from the resulting mature mRNA transcript, potentially resulting in therapeutic de-repression of the downstream pORF. As such, two *BDNF* transcripts were identified that qualified as putative exon skipping targets: transcript v11 (NM_001143811.2) and transcript v14 (NM_001143814.2). These transcripts contained at least three 5ʹ UTR exons, with one or more “skippable” exons. In this context, skippable exons are defined as 5ʹ UTR exons that do not contain the transcription start site or pORF translation initiation site, and that contain at least one uORF initiation codon.

The 5ʹ UTRs of each of these transcripts were cloned upstream of a *Renilla* luciferase gene as described above, and variants generated whereby each skippable exon, or combination of exons, was deleted. All constructs were transfected in HEK293T cells and luciferase activity measured 24 h post transfection.

**Figure 4:**
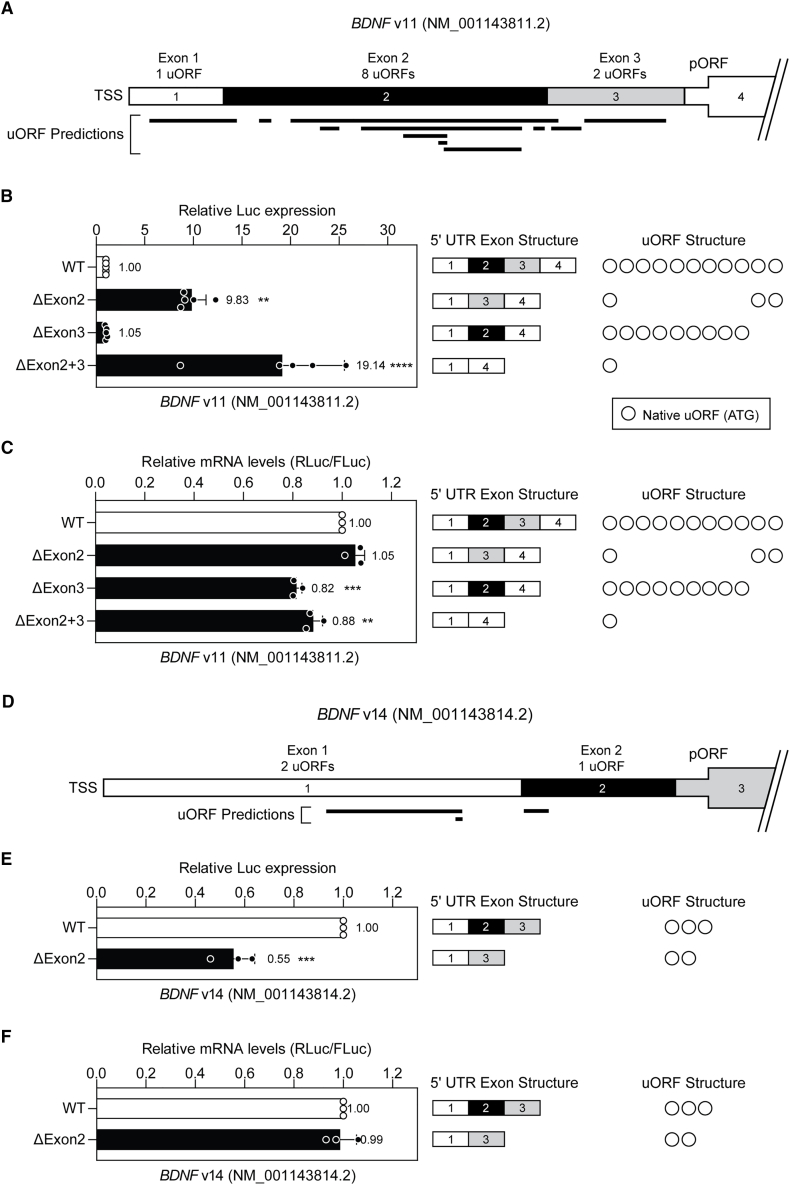
Effect of exon deletion on *BDNF* primary ORF translation (A) Schematic of the 5ʹ UTR for the *BDNF* v11 transcript (NM_001143811.2) indicating the sizes and positions of exons, the locations of predicted uORFs, and the number of uORFs per exon. The pORF start codon is located in exon 4. (B) HEK293T cells were transfected with *BDNF* v11 5ʹ UTR-DLR wild-type and mutant constructs as indicated, and luciferase activity determined after 24 h. For each mutant, the exon structure and number of functional uORFs (open circles) are indicated. (C) RT-qPCR was used to determine RLuc transcript levels normalized to FLuc expression. (D) Schematic of the 5ʹ UTR for the *BDNF* v14 transcript (NM_001143814.2). The pORF start codon is located in exon 3. (E) HEK293T cells were transfected with *BDNF* v14 5ʹ UTR-DLR wild-type and ΔExon2 constructs as indicated, and luciferase activity determined after 24 h (F) RT-qPCR was used to determine RLuc transcript levels normalized to FLuc expression. Values are mean ± SD, *n* = 5 independent experiments for (B) and *n* = 3 independent experiments for (C), (E), and (F). Differences between groups were tested by Student’s *t* test or one-way ANOVA and Bonferroni post hoc test, as appropriate. ∗*p* < 0.05, ∗∗*p* < 0.01, ∗∗∗*p* < 0.001, ∗∗∗∗*p* < 0.0001.

For *BDNF* v11, deletion of exon 2 (containing eight predicted uORFs) resulted in an ∼9.8-fold upregulation of pORF reporter gene expression (*p* < 0.01) (Figures 4A and 4B). Deletion of both exons 2 and 3 resulted in even greater increase in *Renilla* luciferase activity (19.1-fold, *p* < 0.0001). However, deletion of exon 3 alone (containing two predicted uORFs) did not affect pORF reporter gene expression. The profound upregulation in luciferase activity observed could not be explained by changes in transcript levels as there was no difference observed between groups (Figure 4C).

These data suggest that exon skipping strategies that aim to exclude *BDNF* v11 exon 2, or exons 2 and 3, from the mature *BDNF* transcript could potentially be exploited for therapeutic BDNF upregulation at the level of translation.

In contrast, analysis of *BDNF* v14 showed that when exon 2 was deleted (containing one uORF) there was no significant effect on reporter gene expression (Figures 4D and 4E). These data could not be explained by changes in transcript levels, consistent with translation level effects (Figure 4F). Overall, *BDNF* v14 was found to be an unsuitable target for upstream exon skipping.

**Figure 5:**
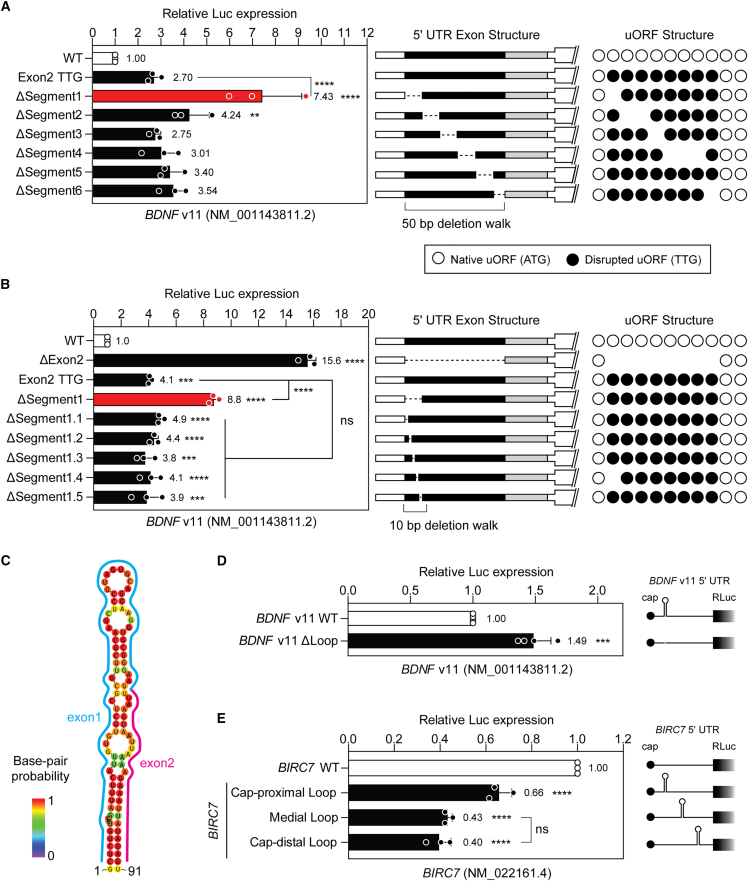
An RNA hairpin structure contributes regulated expression of *BDNF* v11 (A) HEK293T cells were transfected with *BDNF* v11 (NM_001143811.2) 5ʹ UTR-DLR wild-type and mutant constructs as indicated, and luciferase activity determined 24 h post transfection. Constructs were generated containing the four exons of the *BDNF* v11 5ʹ UTR in which all exon 2 uORFs were disrupted by ATG to TTG substitutions, and in which 50 bp regions spanning exon 2 were sequentially deleted. (B) HEK293T cells were treated as above with *BDNF* v11 constructs in which 10 bp regions spanning the first 50 bp of exon 2 were sequentially deleted. (C) Structure of predicted RNA hairpin spanning the exon1-2 boundary. Dual luciferase assay data for plasmid constructs in which the putative hairpin is (D) deleted from the *BDNF* v11 5ʹ UTR, and (E) inserted into an unrelated 5ʹ UTR (of *BIRC7*, NM_022161.4) at positions as indicated. Values are mean ± SD, *n* = 3 independent experiments. Differences between groups were tested by Student’s *t* test or one-way ANOVA and Bonferroni post hoc test, as appropriate. ∗∗*p* < 0.01, ∗∗∗*p* < 0.001, ∗∗∗∗*p* < 0.0001, ns, not significant. Statistical comparisons are to the WT control group unless otherwise indicated.

Based on these findings we selected *BDNF* v11 for further analysis. We hypothesized that the upregulation observed when exon 2 was deleted might be a consequence of the exclusion of eight uORF sequences from the resulting “exon skipped” mRNA. To this end, constructs were tested in which all eight uORFs within exon 2 were disrupted by mutating their start codon ATG trinucleotides to TTG. Disruption of all eight uORFs located in exon 2 resulted in an ∼2.7-fold upregulation in reporter gene expression (Figure 5A), consistent with the relief of uORF-mediated repression. However, this upregulation was only a fraction (∼36%) of that observed when exon 2 was deleted in its entirety, suggesting that while uORFs contribute to pORF repression, additional regulatory elements may be contributing to its translational repression.

We reasoned that other motifs located within might be responsible for the upregulation effect. To investigate this, a motif-finding analysis was performed on the sequence of *BDNF* v11 exon 2 using BRIO (BEAM RNA Interaction mOtifs),^41^ which identified two RNA binding motifs of interest (HuR #1 and HuR #2) (Figure S2A). However, deletion of these motifs, both individually and together, had no significant effect on pORF reporter expression (Figure S2B), suggesting that they are not responsible for the translation repressing activity contained within exon 2.

We next performed deletion walks to identify a nucleotide sequence in the *BDNF* v11 exon 2 that could account for its translation repressive activity. Mutant constructs were generated in which 50 base pair (bp) contiguous regions of exon 2 were sequentially deleted. For the purpose of this experiment, all uORFs within exon 2 were disrupted, such that non-uORF repressive elements could be potentially identified. Deletion of the first 50 bp (ΔSegment 1) resulted in a pronounced 7.4-fold upregulation in reporter activity relative to the control construct where all the uORFs in exon 2 were disrupted (*p* < 0.0001) (Figure 5A). These data suggest that the combination of uORF-mediated repression, together with a further regulatory element contained within these first 50 bp accounts for the majority of the repressive activity of this exon. A second series of deletion mutants were generated whereby 10-bp regions were deleted across the 50-bp region of interest at the start of exon 2. No upregulation activity was observed for any of these constructs (Figure 5B). Based on these data, we postulated that an RNA structural element, rather than a short sequence motif, might be responsible for the repressive activity mediated by the presence of *BDNF* v11 exon 2.

### Analysis of RNA structure in the *BDNF* v11 5ʹ UTR

RNA secondary structures were predicted for the *BDNF* v11 transcript using RNAfold,^42^ together with all of the various deletion constructs. The WT *BDNF* v11 transcript was predicted to contain a prominent secondary structure motif that spanned the exon 1–2 boundary (Figure 5C). This structure consisted of an imperfect 91 nucleotide hairpin with a stem length of 42 nucleotides and a minimum free energy of −23.5 kcal/mol. This structural motif was predicted to be absent when either exon 2 was deleted, or the first 50 bp of exon 2 were deleted but was broadly maintained in other constructs (Figure S3), suggesting that this element may be contributing to *BDNF* v11 translational repression.

Deletion of the hairpin resulted in an ∼1.5-fold de-repression of downstream reporter expression (*p* < 0.001), consistent with it acting as a repressive element (Figure 5D). Notably, the magnitude of upregulation was less than that observed for the smaller deletions described above (Figures 5A and 5B), potentially because larger deletions may be impacting other regulatory signals within the *BDNF* v11 5ʹ UTR. To investigate the capacity of the *BDNF* v11 5ʹ UTR hairpin to induce translational repression in other contexts, we generated constructs in which the hairpin structure was inserted into the 5ʹ UTR of an unrelated gene. The *BIRC7* gene (NM_022161.4) was selected, as its 5ʹ UTR lacked any predicted uORFs. Insertion of the *BDNF* hairpin into the *BIRC7* 5ʹ UTR resulted in significant (*p* < 0.001) repression of downstream reporter expression (Figures 5E and S4). Repression was observed irrespective of whether the hairpin was inserted in the cap-proximal, medial, or cap-distal positions with respect to the 5ʹ cap, although repression was strongest in the medial and cap-distal positions (∼3-fold downregulation). Taken together, these data suggest that a combination of an RNA secondary structure element, uORFs, and potentially other factors, combine to strongly repress the translational output of the *BDNF* v11 transcript.

### Characterization of *BDNF* transcript expression in human brain and brain-derived cell cultures

**Figure 6:**
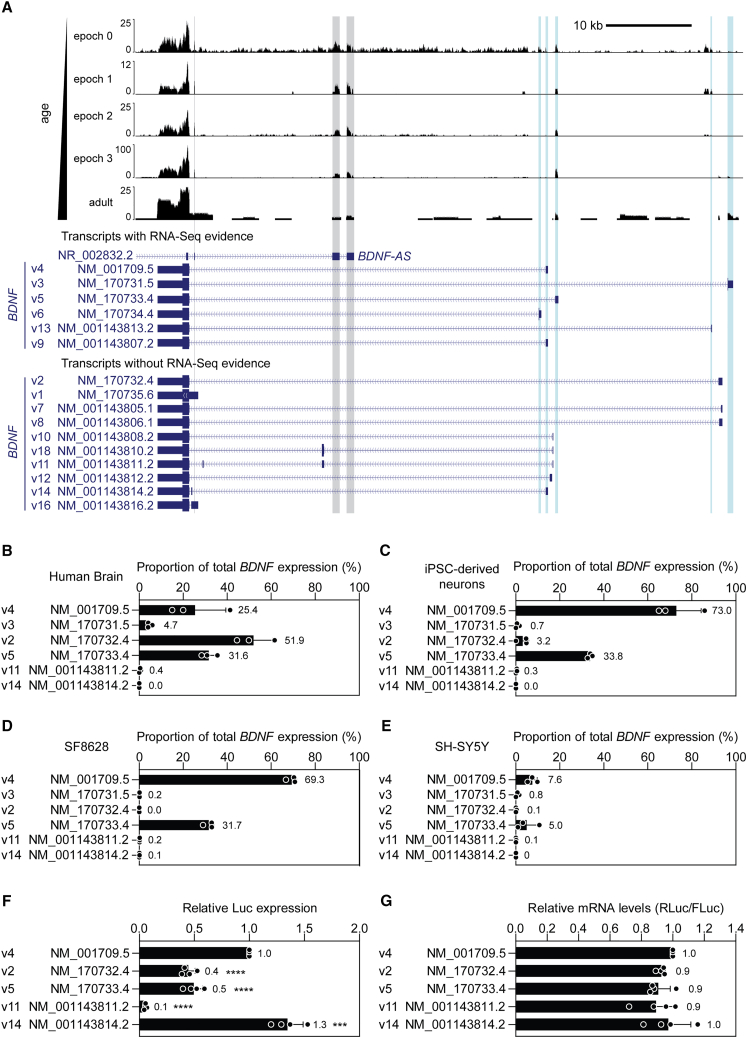
Characterization of *BDNF* transcript isoform expression (A) Publicly available RNA-seq from the developing brain and long-read sequencing data from adult human brain were visualized for the *BDNF* locus. Annotated transcript isoforms were sorted according to whether there is empirical evidence for their expression in human brain. RNA-seq data were aggregated from individual libraries (*N* = 176) that were separated according to developmental epochs (0 = embryonic/fetal [*n* = 9], 1 = fetal [*n* = 83], 2 = late fetal transition/infancy [*n* = 39], 3 = infancy/childhood/adolescence [*n* = 45]). The adult sample is derived from an Alzheimer’s patient brain. Blue vertical bars highlight exons with evidence of expression. Gray bars highlight read density associated with an overlapping antisense long non-coding RNA gene (*BDNF-AS*). Expression of *BDNF* transcripts was determined by RT-ddPCR for (B) human brain (age 30 female), (C) iPSC-derived motor neuron cultures (*n* = 3 lines), (D) SF8628 pediatric diffuse intrinsic pontine glioma cells, and (E) SH-SY5Y neuroblastoma cells. (F) HEK293T cells were transfected with dual luciferase reporter constructs carrying various wild-type *BDNF* promoter variants and luciferase activity assessed 24 h later. (G) RNA was extracted from separate cultures treated in the same way and the ratio of *Renilla* and firefly luciferase transcripts determined by RT-qPCR. Values are mean ± SD. Differences between groups were tested by one-way ANOVA and Bonferroni post hoc test, ∗∗∗*p* < 0.001, ∗∗∗∗*p* < 0.0001. *n* = 3 healthy cell lines for iPSC data. *n* = 3 technical replicates for other cell lines and human brain RNA data. *n* = 4 independent experiments for luciferase and RT-qPCR data.

We next aimed to determine whether the transcript isoforms investigated above are expressed in human brain via qualitative inspection of publicly available RNA-seq data from the developing human brain (covering transitions from embryonic, fetal, infancy, and adolescence stages)^43^ and long-read RNA-seq data from the brain of an adult Alzheimer’s disease patient. RNA-seq read density was observed at 5ʹ UTR exons for RefSeq transcripts: NM_001709.5, NM_170731.5, NM_170733.4, NM_170734.4, NM_001143813.2, and NM_00143807.2 (also known as *BDNF* transcript variants v4 (MANE), v3, v5, v6, v13, and v9, respectively, Figure 6A). Detection of some of these transcript variants was experimentally confirmed in human brain RNA by reverse transcriptase-droplet digital PCR (RT-ddPCR), although the most highly abundant transcript isoform was found to be NM_170732 (v2) in contrast with the RNA-seq data (Figure 6B).

The same RT-ddPCR assays were applied to a variety of human cell models including iPSC-derived motor neurons (Figure 6C), SF8628 (Figure 6D), and SH-SY5Y cells (Figure 6E). The most highly abundant transcript isoform was found to be the MANE variant (v4) in these cells, followed by NM_170733 (v5). Notably, the “skippable” *BDNF* transcripts were expressed at very low, or undetectable levels, in both human brain RNA and total RNA from the various cell models, meaning that these transcripts are unlikely to be suitable targets for therapeutic BDNF upregulation.

We next compared the translational activity of the various WT 5ʹ UTRs previously investigated (Figure 6F) and compared these to the relative luciferase output from the MANE variant (NM_001709, v4). Interestingly, expression was reduced by ∼60% in variants NM_170732 (v2) and NM_170733 (v5) (*p* < 0.0001), suggesting that while NM_170732 may be expressed at higher levels than the MANE variant in human brain, it exhibits a lower level of translational activity.

*BDNF* v11 (NM_001143811) was found to exhibit ∼90% less expression than the MANE variant (*p* < 0.0001) (Figure 6F), consistent with our observations that this transcript has high potential for upregulation via upstream exon skipping (Figure 4B). However, the low levels of expression of this transcript preclude this as a potential therapeutic target in human brain (Figures 6A–6E).

Interestingly, *BDNF* v14 (NM_001143814) was found to be ∼30% more translationally active than the MANE variant (*p* < 0.001). This finding suggests that low abundance transcripts (such as NM_001143814) may nevertheless exhibit high translational activity (Figure 6F). The skipping of exon 2 in *BDNF* v14 effectively results in the generation of the MANE variant *BDNF* v4, and so this result is consistent with the results shown in (Figure 4E). Luciferase transcript levels in cultures transfected with the various WT 5ʹ UTR constructs were not significantly changed (Figure 6G), suggesting that the differences in luciferase activity observed between 5ʹ UTR constructs occur at the level of translation.

A study by Esvald et al. recently conducted an extensive characterization of *BDNF* transcript expression in human brain.^44^ In this study, transcript isoform expression was determined by counting reads mapping to unique 5ʹ UTR exons. Broadly speaking, data for dorsolateral prefrontal cortex samples were similar to our findings. Transcript variants *BDNF* v11 and *BDNF* v14 were not expressed, whereas transcript variants *BDNF* v2, *BDNF* v3, and *BDNF* v4 were consistently expressed.^44^ In contrast with our findings, Esvald et al. observed that the MANE Select transcript *BDNF* v4 was the most abundant transcript variant. Additionally, we observed expression of *BDNF* v5, which was mostly absent in the Esvald et al. data.^44^

### BDNF protein upregulation via base editing-mediated disruption of a uORF start codon

**Figure 7:**
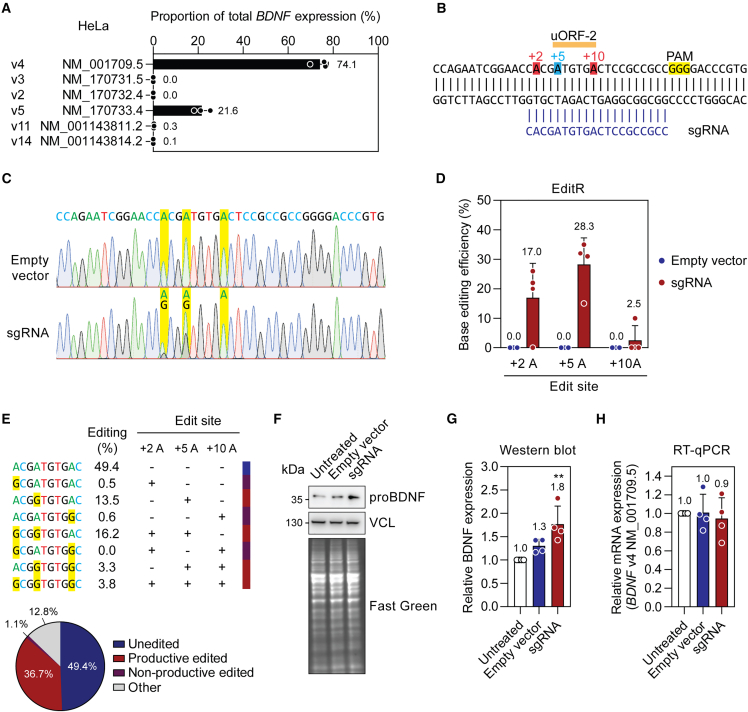
Upregulation of BDNF protein following base editing-mediated disruption of a uORF start codon (A) Expression of *BDNF* transcript isoforms was determined by RT-ddPCR for HeLa cells. (B) Schematic of CRISPR-base editing strategy targeting the start codon of the *BDNF* v4 uORF-2. HeLa cells were transfected with plasmids encoding a nickase SpCas9 fused to an adenosine deaminase (ABE8e) and a single-guide RNA (sgRNA). Cells were dissociated 3 days post transfection, counted, and re-seeded at a defined cell density. Materials was collected for further analysis after an additional 12 h. (C) Representative Sanger sequencing chromatograms for control (Empty vector) and on-target sgRNA-treated cells. The target adenosine and potential bystander adenosines in the base editing window are highlighted in yellow. (D) Quantification of adenine base editing by EditR analysis. (E) Amplicon-seq analysis quantifying on-target and bystander editing events. (F) Representative western blot image showing BDNF protein detection. Equal protein loading was determined by blotting for vinculin (VCL) and by total protein staining using Fast Green. (G) Western blot quantification. (H) RT-qPCR analysis of *BDNF* transcript levels, normalized to *ACTB* expression. Values are mean ± SD, *n* = 4 independent experiments. Differences between groups were tested by one-way ANOVA and Bonferroni post hoc test, ∗∗*p* < 0.01.

Based on the uORF validation data (Figure 2B) and experimentally observed transcript expression patterns in human brain and brain-derived cell models (Figure 6), we selected uORF-2 in *BDNF* v4 for further investigation. To maximize the chance of experimental success, we used HeLa cells to test experimental uORF manipulation approaches based on their relative ease of transfection. The *BDNF* v4 transcript was found to be the major expressed isoform in HeLa cells (Figure 7A), exhibiting a pattern of expression that was similar to that observed for iPSC-derived motor neurons (Figure 6C).

A CRISPR-nCas9 adenine base editing strategy using ABE8e^45^ was devised, targeting *BDNF* v4 uORF-2 (Figure 7B). HeLa cells were transfected with plasmids encoding ABE8e and a single-guide RNA (sgRNA) targeting uORF-2. Cells were harvested after 3 days and re-seeded at a consistent cell density across groups. In parallel, DNA editing was assessed by Sanger sequencing. After a further 12 h, cells were harvested for protein and RNA analyses. Untreated cells, and cells treated with ABE8e and an empty sgRNA vector plasmid (empty vector) served as negative controls. Successful base editing was apparent from inspection of Sanger sequencing chromatograms, with overlaid A and G peaks observed at the target +5 site. Notably, some bystander editing was apparent at the +2 site, but not at the +10 site (Figure 7C). Quantification of the editing using EditR^46^ revealed a mean target editing of 28.3% (with a maximum of 35%) (Figure 7D). Mean bystander editing was quantified as 17% at the +2 site and 2.5% at the +10 site. No base editing was observed in empty vector-treated cultures. The sample with the highest on-target base editing efficiency was selected for further analysis by next-generation amplicon sequencing. This sample was selected in order to best capture the most diversity in editing outcomes. Quantification of editing outcomes revealed that productive editing outcomes (where the uORF-2 ATG was edited) represented 36.7% of all reads (Figure 7E), which closely matched the Sanger sequencing quantification (Figure 7C). Non-productive editing (whereby bystander editing occurred without uORF ATG editing) was observed in 1.1% of reads. Target uORF editing was associated with an ∼1.8-fold (*p* < 0.01) increase in BDNF protein (pro form) expression by a maximum of 2.3-fold, consistent with partial uORF disruption (Figures 7F and 7G). *BDNF* v4 mRNA levels were not significantly changed by the treatment, suggesting that increased proBDNF protein levels are due to changes at the translational level, consistent with uORF disruption (Figure 7H). No DNA editing or BDNF protein upregulation was observed using a non-targeting sgRNA or when the on-target sgRNA was combined with a cytosine base editor construct (Figure S5).

**Figure 8:**
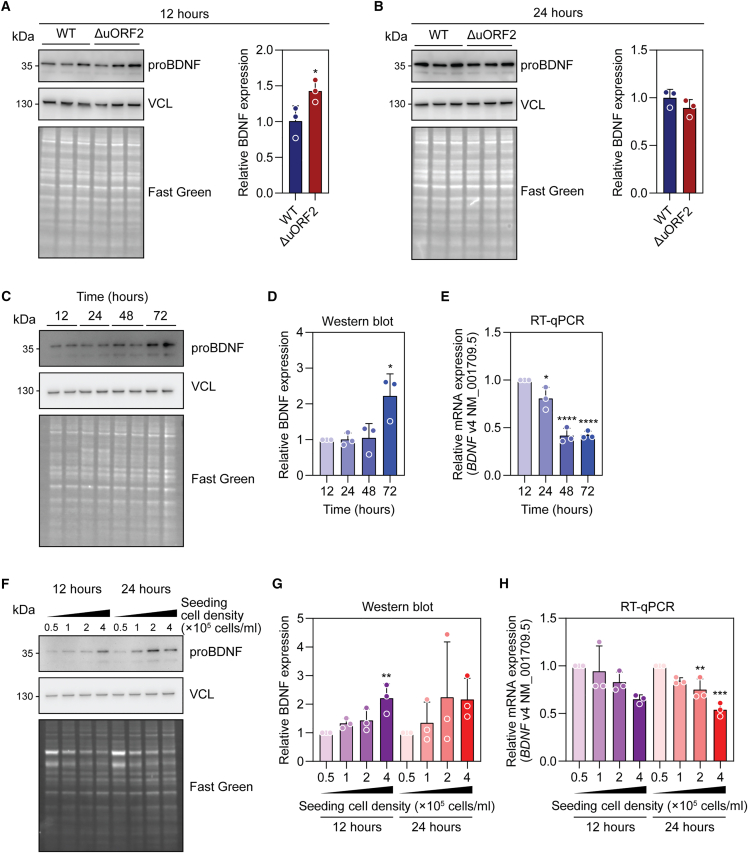
Investigation of uORF-disruption-mediated BDNF upregulation in edited HeLa cell clones Isolated HeLa cell clones carrying a uORF disrupting single base edit (ΔuORF2) were propagated, seeded, and harvested at (A) 12 h, or (B) 24 h. BDNF protein expression determined by western blot. Unedited clones containing the wild-type (WT) *BDNF* uORF served as controls. WT HeLa cells were seeded and lysates collected 12, 24, 48, and 72 h later. (C) BDNF expression was determined by western blot (with duplicate well technical replicates), and (D) quantified by densitometry. (E) Separate cultures were analyzed by RT-qPCR to measure *BDNF* v4 transcript levels normalized to *ACTB* expression. WT HeLa cells were seeded at starting cell densities of 0.5, 1, 2, and 4 ×10^5^ cells/mL and lysates harvested 12 or 24 h later. (F) BDNF expression was determined by western blot, and (G) quantified by densitometry. (H) Separate cultures were analyzed by RT-qPCR as described above. Values are mean ± SD. For (A) and (B), differences between groups were assessed by one-tailed Student’s *t* test, *n* = 3 separate clones, each reported as the mean of *n* = 2–3 independent experiments. For (C)–(H), differences between groups were tested by one-way ANOVA and Bonferroni post hoc test. *n* = 3 independent experiments, ∗*p* < 0.05, ∗∗*p* < 0.01, ∗∗∗*p* < 0.001, ∗∗∗∗*p* < 0.0001.

Clonal HeLa lines containing the desired *BDNF* v4 uORF-2 (ΔuORF2), and non-edited control clones (*n* = 3), were obtained by limiting dilution. proBDNF protein was significantly (*p* < 0.05) upregulated in the ΔuORF2 lines by 1.43-fold when lysates were collected 12 h after seeding (Figure 8A) but not after 24 h (Figure 8B). We reasoned that the effects of uORF disruption may be being overridden by another BDNF regulatory mechanism. To test this, WT HeLa cells were collected at 12, 24, 48, and 72 h after seeding. proBDNF expression was significantly (*p* < 0.05) upregulated by 2.2-fold at 72 h (Figures 8C and 8D), whereas *BDNF* v4 transcript levels exhibited a reciprocal pattern of progressive downregulation (Figure 8E). These data suggested that cell crowding might have an effect on BDNF expression that is overwhelming the uORF regulation effect. To test this hypothesis, WT HeLa cells were seeded at four different densities (0.5, 1, 2, and 4 × 10^5^ cells/mL) and cells harvested either 12 or 24 h later. In the case of both time points, a progressive increase in BDNF protein expression was observed (Figures 8F and 8G), while the opposite effect was observed for *BDNF* v4 transcript levels. Taken together, these data show that in cycling HeLa cells, *BDNF* expression is regulated in a cell density-dependent manner, and that the magnitude of this effect is sufficient to overwhelm the upregulation effect observed following targeted disruption of *BDNF* v4 uORF-2.

## Discussion

The *BDNF* gene exhibits a complex transcriptional landscape with the potential to generate multiple transcript isoforms. The majority (14 out of 17 RefSeq annotated transcripts) are predicted to contain at least one uORF (Figure 1; Table S1). Five *BDNF* transcripts were selected for experimental characterization, all of which were validated as being uORF-regulated to some extent, suggesting that uORF regulation plays an important role in shaping the translational output of the *BDNF* locus (Figures 2, 3, 4, and 5). However, the pattern of uORF regulation differed between transcripts, and a number of interesting features were observed. For transcripts containing multiple uORFs, we typically observed additive effects (Figures 2 and S1). We also observed redundancy between uORF activity (Figures S1 and 3), and that not all uORFs are equal, with some exhibiting a greater contribution to pORF repression than others (Figures 2 and S1). Moreover, we observed that the minimal uORF (i.e., methionine-stop) is capable of functioning as a repressive uORF (Figure 2).

The multitude of uORF-regulated transcripts originating from the *BDNF* locus present exciting potential opportunities for therapeutic manipulation. It has previously been reported by Liang et al. that uORF activity can be disrupted to activate pORF translation using steric block antisense oligonucleotides (ASOs) targeted to uORF start codons.^47^ There have since been conflicting reports regarding the capability of ASOs to interfere with uORF function. Our group has been unable to reproduce some of the main findings from this study by targeting the *RNASEH1* uORF using the same ASO sequences reported by the authors.^48^ Nevertheless, some studies have reported similar effects targeting 5ʹ UTRs with steric block antisense oligonucleotides.^49^^,^^50^^,^^51^ Importantly, some studies have reported uORF-independent protein upregulation effects with ASOs overlapping a uORF in the *SMN2* 5ʹ UTR^52^ and targeting non-uORF sequences in the *FXN* 5ʹ UTR,^53^ in both cases pORF upregulation effects were attributed to mRNA stabilization mechanisms. Interestingly, Hedaya et al. have employed steric block ASOs in a distinct manner to modulate uORF function.^54^ Specifically, ASOs can be used to disrupt crosstalk between RNA secondary structure elements and uORF translation initiation events that either enhance or diminish pORF translational output.^54^ The authors showed that secondary structure-disrupting ASOs can counteract cardiac hypertrophy in various animal model systems by promoting GATA4 protein expression.^54^ Importantly, this study demonstrated that ASOs that directly target uORF start codons can result in inhibition of pORF translation,^54^ in contrast to the report of Liang et al. and in line with findings from our group.^47^^,^^48^ The complexities surrounding direct targeting of uORF start codons with steric block approaches motivated us to explore alternative approaches for therapeutic uORF interference.

Upstream exon skipping is a particularly attractive approach for counteracting the repressive effects of uORFs. There are multiple Food and Drug Administration-approved splice switching oligonucleotides (in non-uORF contexts), suggesting that this modality has reached a point of maturity.^55^ Most notably, Nusinersen (Spinraza) is a highly effective splice switching oligonucleotide for the treatment of spinal muscular atrophy, which is an 18-mer composed of full phosphorothioate-2ʹ-*O*-methoxyethyl chemistry that is administered by the intrathecal route. The example of Nusinersen has shown that this pattern of oligonucleotide chemistry and route of administration are viable approaches for the long-term treatment of infants and children. The widespread distribution of Nusinersen throughout the CNS is highly encouraging for the treatment of brain indications, as would be required for *BDNF*-targeted therapies.^56^ As such, Nusinersen has served as a “template” for other therapies. Most notably, Milasen was developed as a personalized splice switching therapy for a single patient with CLN7 Batten disease modeled on Nusinersen.^57^ Similarly, the Nusinersen template might easily be re-deployed to skip upstream 5ʹ UTR exons with the aim of disrupting uORF function. To this end, we explored two *BDNF* variants with “skippable” upstream exons (Figure 4). Deletion of exon 2 of *BDNF* v11 resulted in a profound upregulation effect, thereby validating this approach. However, inspection of RNA-seq data from developing cortex, together with absolute quantification RT-ddPCR in adult human brain and brain-derived cell lines suggests that this transcript is present at very low levels or not detecable (Figure 6). As such, there is no transcript present in which to achieve exon skipping. Furthermore, were splice manipulation possible in a minority constituent transcript (i.e., *BDNF* v11), the effect on downstream protein expression is likely to be imperceptible against a background of BDNF protein derived from other, more abundant, *BDNF* transcripts. However, such an upstream exon skipping approach is likely to be applicable in other genetic/therapeutic contexts (e.g., for the treatment of neurodevelopmental haploinsufficiency disorders). Notably, similar effects has been reported in the context of uORF regulation of *CD20*^58^ and *RMBXL1*.^59^ Additionally, we found that other factors, such as the presence of RNA structural motifs, might also be disrupted through upstream exon skipping, expanding the possible utility of this approach.

By contrast, deletion of exon 2 in *BDNF* v14 resulted in a slight decrease in reporter expression. These findings demonstrate that while the removal of one or more exons has the potential to exclude negative regulatory information from the mature transcript, it might equally remove positive regulatory information, leading to a decrease in target protein expression. Interestingly, skipping of *BDNF* v14 exon 2 effectively converts this transcript isoform into another annotated isoform, *BDNF* v4. We have demonstrated that this transcript contains a potent uORF in exon 1, and that skipping of exon 2 decreases the intercistronic distance between the uORF and pORF, which may account for the observed increase in pORF repression.^60^^,^^61^ These findings, which are not immediately obvious, suggest that potential uORF therapeutic targets must be functionally validated to confirm the intended direction of effect. Furthermore, our data highlight the importance of validating the presence of the target transcript in disease-relevant tissues, as considerable variability in exon inclusion and transcription start site usage is often observed at gene 5ʹ regions.

We next sought to use a base editing strategy for targeted uORF manipulation. Transient transfection of HeLa cells with the ABE8e adenine base editor and a uORF-targeting guide RNA resulted in up to 35% editing and a corresponding ∼1.8-fold activation of BDNF protein expression in transiently transfected cells (Figure 7) and ∼1.4-fold activation in clonally isolated cultures. These data provide proof-of-concept evidence that uORFs can be manipulated for the purpose of upregulation in the specific case of *BDNF* (Figure 8). Interestingly, when cells were cultured for prolonged periods, the effects of uORF-disruption-mediated BDNF upregulation were lost, which we attribute to a cell density-associated upregulation of BDNF expression that overwhelmed the uORF effect (Figure 8). This aspect of BDNF regulation must be carefully considered when advancing this approach in more physiologically relevant cell models and small animal studies.

Importantly, editing takes place in non-protein-coding regions, where off-target effects are expected to have limited impact. An exciting possibility is that such an approach would have broader applicability, especially considering the abundance of predicted uORFs in the human transcriptome. Indeed, CRISPR-Cas9-mediated uORF disruption has been reported in plants,^62^^,^^63^ and in human cells.^64^ More recently, a CRISPR-Cas13d-TadA8e fusion system was used to edit a disease-causing uORF in the *IRF6* gene via RNA adenosine-to-inosine editing using a reporter system.^65^

An obvious extension of the approach described here would be to use RNA editing technologies for transient targeting of a *BDNF* uORF. A variety of oligonucleotide triggers have been proposed with various designs and chemical compositions.^66^^,^^67^^,^^68^ Such a technology would be in many ways preferable, as editing trigger oligonucleotides could be introduced via intrathecal injection, with the degree of target protein upregulation tuneable and/or reversible through the control of dosing. Similarly, expressed RNA editing triggers have also been described,^69^ which may have similar upregulation potential if deployed appropriately.

Further therapeutic translation of *BDNF* targeting strategies will likely necessitate the use of rodent models, and thereby modification of the effector molecules will be needed so that they can target the corresponding mouse or rat sequences. Alternatively, small regions of the rodent 5ʹ UTR could be humanized in order to enable further translational development. It will also be important to determine the functional consequences of *BDNF* uORF interference in more physiologically relevant models. Notably, the high sensitivity to the effects to cell confluence may present an obstacle to further therapeutic development.

In summary, this study identified *BDNF* as a promising target for uORF manipulation therapies. We have characterized uORF-mediated *BDNF* transcript regulation and described two BDNF upregulation approaches. Using base editing we were able to demonstrate robust BDNF protein activation even after transient transfection and ∼28% editing at the target uORF start codon adenosine. To our knowledge, this is the first study to describe uORF-mediated disruption via adenine base editing. Such base editing approaches could, in principle, be applied to a myriad of other potential uORF targets beyond *BDNF* and would offer several advantages over existing approaches. In particular, adenine base editing provides a permanent modification (at least for the lifetime of the edited cell) and is not dependent on the existence of specific 5ʹ UTR exon configurations or regulatory secondary structures.

A limitation of the current study is its focus on a single gene. Understanding the complexities of uORF regulation at the *BDNF* locus has required substantial effort, and importantly, scaling such a base editing approach to other gene targets may require similar levels of biological insight. Future studies must confirm expression of the target transcript and consider the relevant isoform expression context, ideally supported by improved pipelines for transcript annotation and isoform-aware target selection. In addition, sequence conservation between human and small animal models is an important consideration for therapeutic translatability. This may include prioritization of targets with high conservation, or where appropriate, developing strategies to generate humanized mouse models (as described above).

Given the high prevalence of uORFs in the transcriptome and the potential for base editing technologies to induce edits at almost any compatible sequence, it is likely that this strategy will have broad applicability beyond *BDNF*. This study provides a template for the application of these technologies for the upregulation of other therapeutic target genes.

## Materials and methods

### Bioinformatics and public datasets

uORFs were predicted using custom in-house software. Ribo-seq data and RNA-seq data were obtained from GWIPS-viz.^70^^,^^71^ Visualization of Ribo-seq/RNA-seq data in RNA space was performed using custom in-house software. Details of *BDNF* transcript isoforms were downloaded using the Table Browser function of the UCSC Genome Browser.^72^ RNA-seq data from the BrainVar consortium was used to determine *BDNF* transcript isoform expression.^43^ These data are rRNA-depleted (by ribozero) bulk tissue libraries derived from human dorsolateral prefrontal cortex (*N* = 176). Data were stratified by developmental stage (i.e., epoch). Epoch 0: first trimester, epoch 1: second trimester, epoch 2: third trimester and infancy, and epoch 3: infancy to adult. Long-read RNA-seq data (PacBio platform) from an adult brain from an Alzheimer’s disease patient were obtained from the PacBio website (https://downloads.pacbcloud.com/public/dataset/Alzheimer2019_IsoSeq/). Motif-finding analysis was performed using BRIO.^41^ RNA structure analysis and visualization was performed using RNAfold.^42^

### Human brain RNA

Human brain RNA was obtained from Thermo Fisher Scientific (product no: AM7962, lot no: 2661603). The sample was obtained from a 30-year-old Caucasian female (cause of death: anoxia/asphyxiation).

### Reporter plasmid generation

Plasmid constructs were generated in-house by Gibson assembly or generated as a service by Azenta Life Sciences (Manchester, UK). A custom-generated dual luciferase reporter was used for uORF validation. This vector consists of two independent reporter transgene cassettes: firefly luciferase (FLuc) and *Renilla* luciferase (RLuc).

### Cell culture

All immortalized cells (HEK293T, SH-SY5Y, SF8628, and HeLa) were cultured in a humidified incubator at 37°C (5% CO_2_) in Dulbecco’s modified Eagle’s medium (DMEM-Glutamax) that was supplemented with 10% fetal bovine serum (FBS) (both Thermo Fisher Scientific) and 1% penicillin, streptomycin and amphotericin B (PSA) (Merck Life Science, Gillingham, UK). Cell cultures were confirmed free of mycoplasma contamination through monthly testing.

For reporter construct assays, cells were transfected with plasmids using Lipofectamine 2000 (Thermo Fisher Scientific) or polyethylenimine (PEI). For Lipofectamine 2000 transfection, plasmids and transfection reagent were prepared separately in Opti-MEM before being combined, incubated for 10 min, and then added to cells in a dropwise manner, according to the manufacturer’s instructions. A ratio of 1 μL of Lipofectamine 2000 to 1 μg of plasmid DNA was used. For PEI transections, a weight:weight ratio of 4:1 transfection reagent:plasmid DNA was used. PEI and plasmid DNA were prepared separately in DMEM, combined, and then incubated for 20 min, before dropwise addition to cells. A total of 100 ng or 500 ng plasmid DNA was used for 96-well and 24-well plates, respectively.

For base editing experiments, transfection was performed using Lipofectamine 2000 with a ratio of 2.1 μL transfection reagent per 1 μg of plasmid DNA. A total of 2.5 μg of plasmid was transfected per well of a 24-well plate.

### iPS cells

The iPSC lines used in this study were derived from skin biopsy fibroblasts, collected under ethical approval granted by the South Wales Research Ethics Committee (WA/12/0186) in the James and Lillian Martin School Center for Stem Cell Research, University of Oxford, under standardized protocols that we have described elsewhere.^73^ Fibroblasts and derived iPSC lines tested negative for mycoplasma (MycoAlert, Lonza, UK). The iPS cells were differentiated into motor neurons *in vitro* according to our previously published methods.^73^ Briefly, the iPS cells were cultured on Geltrex in mTESR 1 supplemented with mTESR supplement (both Stem Cell Technologies, Cambridge, UK) and antibiotics (Thermo Fisher Scientific). Induction media was added to the iPS cells (at 90% confluence) and consisted of DMEM/F12/Neurobasal medium 1:1, 1× N2 supplement, 1× B27 supplement (all Thermo Fisher Scientific), ascorbic acid (0.5 μM, Sigma-Aldrich, Gillingham, UK), β-mercaptoethanol (50 μM, Thermo Fisher Scientific), Compound C (1 μM, Bio-Techne, Abingdon, UK), Chirr99201 (3 μM, Bio-Techne). On the second day, the induction medium was supplemented with all-*trans* retinoic acid (1 μM, Sigma-Aldrich) and Smoothened Agonist (500 nM, Bio-Techne). Two days later, Chirr99201 and Compound C were removed from the medium. On the ninth day of differentiation, neural progenitor cells were split 1:3 using Accutase (Thermo Fisher Scientific) and ROCK inhibitor (Bio-Techne) was added for 24 h. On day 19, the progenitors were split in their final plating conditions and the medium was supplemented with BDNF (10 μM, Thermo Fisher Scientific), GDNF (10 μM, Thermo Fisher Scientific), and laminin (500 ng/μL). DAPT (10 μM, Bio-Techne) and ROCK inhibitor were added for the first 7 days, and then removed for the remainder of the maturation. RNA was collected between days 30 and 35.

### Dual luciferase assay

Luciferase activity was determined using the Dual-Glo Luciferase assay (Promega) according to manufacturer’s instructions. Briefly, HEK293T cells were transfected with plasmids as appropriate in quadruplicate. Twenty-four hours after transfection, half of the media volume was removed, and Dual-Glo Reagent was added to each well. Samples were incubated at room temperature for 10 min and then the cell lysates transferred to a flat-bottomed Greiner 96-well plate (Merck). FLuc activity was measured, followed by quenching with an equal volume of Stop & Glo Reagent, a second 10-min incubation at room temperature, and then measurement of RLuc activity. Luciferase activities were recorded on a CLARIOstar luminometer (BMG Labtech, Aylesbury, UK) using a focal height of 9.0 mm and the no-filter setting. The *Renilla* luciferase activity was normalized to the firefly luciferase activity for each sample, and the data were scaled such that the mean value of the control group was returned to a value of 1.

### RT-qPCR

To quantify firefly and *Renilla* luciferase transcripts, total RNA was first extracted using the Maxwell RSC Instrument and the Maxwell RSC simplyRNA Tissue Kit (both Promega). Reverse transcription was performed using the high-capacity cDNA reverse transcription kit (Thermo Fisher Scientific) according to the manufacturer’s instructions using 200–1,000 ng (typically 400 ng) input total RNA.

cDNA was diluted 1:5 in nuclease-free water prior to quantitative polymerase chain reaction (qPCR) amplification using Power SYBR Green PCR Master Mix and the StepOnePlus Real-Time PCR System (both Applied Biosystems, Warrington, UK). Universal cycling conditions were used (i.e., 95°C for 10 min, followed by 40 cycles of 95°C for 15 s and 60°C for 1 min) and samples were run in duplicate; 10 pmol of each primer was included per reaction. Reaction specificity was confirmed by post-run melting curve analysis. Relative quantification was performed using the Pfaffl method.^74^ Primer sequences are listed in Table S2.

### RT-ddPCR

Absolute quantification of RNA transcript isoforms was determined by reverse transcription-droplet digital PCR (RT-ddPCR), using diluted cDNA (1:5, prepared as described above). Reactions were prepared using QX200 ddPCR EvaGreen Supermix (Bio-Rad) and 10 pmol of each primer per 20-μL reaction. Droplets were prepared using the QX200 Droplet Generator and QX200 Droplet Generation Oil for EvaGreen (both Bio-Rad). Subsequently, droplets were transferred to a 96-well plate and the plate sealed using the PX1 PCR Plate Sealer (Bio-Rad). PCR was performed using the following cycling conditions: 95°C enzyme activation step for 5 minutes followed by 40 cycles of a two-step cycling protocol (95°C for 30 seconds and 60°C for 1 minute). The ramp rate between these steps was set to 2°C per second. After thermal cycling, the plate was analyzed using a QX600 droplet reader (Bio-Rad) according to the manufacturer’s instructions.

### Base editing

Base editing guide RNA sequences were designed manually by inspection of the sequence and SpCas9 PAM sequences (NGG) identified. The nSpCas9-ABE expression cassette was derived from the ABE8e plasmid^45^ (Addgene, #138489) and cloned into a custom expression vector. Guide RNAs were cloned into a custom U6 promoter expression vector. The sequence of the *BDNF* v4 uORF targeting sgRNA is 5ʹ-CACGATGTGACTCCGCCGCC-3ʹ, and the non-targeting control is 5ʹ-GAACAGCTCTGAACGAGACCC-3ʹ.

To assess base editing efficiency, genomic DNA was extracted using the Maxwell RSC Cell DNA Purification Kit (Promega) following the manufacturer’s instructions. A region of the *BDNF* locus encompassing the edit site was amplified by PCR using Q5 High-Fidelity DNA Polymerase (New England Biolabs) and subject to Sanger sequencing (Azenta Life Sciences). Primer sequences for editing analysis are listed in Table S2. The proportion of edits was quantified using EditR.^46^

Next-generation amplicon sequencing was performed using the Amplicon-EZ service (Azenta Life Sciences). Sequencing reads were processed using custom python scripts that used string matching to identify edits occurring at the target uORF site together with bystander edits. Reads that were not matched using these criteria were classified as “other,” and were either of atypical length or contained additional mismatches in non-target regions of the sequence.

### Western blot

Cells were washed with PBS, and then lysed in RIPA buffer (Thermo Fisher Scientific) with cOmplete EDTA-free protease inhibitor (Roche, Welwyn Garden City, UK). The lysates were cleared by centrifugation at 12,000 × *g* for 10 min at 4°C. Protein concentrations were determined by BCA Protein Assay (Thermo Fisher Scientific) according to the manufacturer’s instructions. Equal amounts of total protein were loaded onto precast 10% NuPAGE Bis-Tris mini or midi gels (Thermo Fisher Scientific) and separated by sodium dodecyl sulfate-polyacrylamide gel electrophoresis (SDS-PAGE). Protein samples were electrotransferred onto a 0.2 μm PVDF (polyvinylidene fluoride) membrane (Merck Millipore, Watford, UK). Membranes were stained with Fast Green FCF (Sigma-Aldrich) and total protein imaged using the ChemiDoc MP Imaging System (Bio-Rad). Membranes were subsequently blocked with 5% milk in Tris-buffered saline supplemented with Tween 20 (TBST). Blocked membranes were incubated with primary antibodies overnight in 2% bovine serum albumin (BSA) in TBST at 4°C. The next day, membranes were washed three times with TBST (10 min each), followed by incubation with secondary antibodies for 1 h at room temperature. Membranes were washed three times and signal developed using Clarity Western ECL Substrate (Bio-Rad). Blots were visualized using the ChemiDoc MP Imaging System. Details of antibodies are provided in Table S3.

### Statistical analysis

Statistical analyses were performed using GraphPad Prism Software Version 10.1.2 (GraphPad Software Inc., San Diego, CA, USA). For comparisons of two samples, a Student’s *t* test was used. For comparisons of more than two groups, an ordinary one-way analysis of variance (ANOVA) was performed with Bonferroni’s post hoc test for inter-group comparisons.

## Data availability

All data are included in the manuscript. Raw data are available on request.

## Acknowledgments

This work was supported by grants from Great Ormond Street Hospital Sparks Fund/10.13039/100012012Dravet Syndrome UK (awarded to M.J.A.W. and T.C.R.), the Oxford University Press John Fell Fund, and Medical Life Sciences Translational Fund (awarded to T.C.R.).

## Author contributions

T.C.R., N.S., B.H., D.G., and M.J.A.W. conceived the study. T.C.R., D.G., and M.J.A.W. supervised the work. N.F., T.G., N.S., D.Y., H.J.F., and B.H. performed experimentation. A.L. and S.J.S. provided informatics analysis. R.D. and K.T. provided iPSC material. T.C.R. wrote the first draft of the manuscript. All authors contributed to the final version of the manuscript.

## Declaration of interests

T.C.R., M.J.A.W., and B.H. have filed a patent related to a uORF-targeting antisense oligonucleotide technology. T.C.R., D.G., N.F., D.Y., and M.J.A.W. have filed a patent related to uORF-mediated BDNF upregulation. T.C.R., M.J.A.W., N.S., and B.H. are founders and shareholders in Orfonyx Bio Ltd, a biotechnology spin-out company that aims to use uORF-targeting technologies for therapeutics development. N.S. is an employee of Orfonyx Bio. T.C.R. and M.J.A.W. are consultants for Orfonyx Bio.

## Supplemental information

Document S1. Figures S1–S5 and Tables S1–S3

Document S2. Article plus supplemental information
