# Supplementary material for: Targeted BDNF upregulation via upstream open reading frame disruption
Source: Mol Ther. 2025 Dec 11;34(3):1652–71. doi: 10.1016/j.ymthe.2025.12.024 (PMC12974206; doi:10.1016/j.ymthe.2025.12.024)
Supplement: Document S1. Figures S1–S5 and Tables S1–S3 [file mmc1.pdf]

YMTHE, Volume *34*

Supplemental Information

Targeted BDNF upregulation via upstream open reading frame disruption

Ning Feng, Thomas Goedert, Nenad Svrzikapa, Dongnan Yan, Hans J. Friedrichsen, Britt Hanson, Alicia Ljungdahl, Ruxandra Dafinca, Kevin Talbot, Stephan J. Sanders, Dhanu Gupta, Mathew J.A. Wood, and Thomas C. Roberts

Supplemental Material

Table S1

BDNF RefSeq transcript variants.

Transcript variants investigated in this study are highlighted in bold.

RefSeq ID	Transcript Variant	Other Names	Encoded protein isoform	Predicted uORFs	5' UTR exons	uORF/exon structure	CDS size (aa)
NM_170735.6	v1	IX	a	22	1	[22]	248
**NM_170732.4**	**v2**	**IIc / BDNF2C**	**a**	**1**	**2**	**[1,0]**	**248**
NM_170731.5	v3	I / BDNF1	b	3	1	[3]	256
**NM_001709.5**	**v4**	**VIb / BDNF5**	**a**	**2**	**2**	**[2,0]**	**248**
**NM_170733.4**	**v5**	**IV / BDNF4**	**a**	**5**	**2**	**[5,0]**	**248**
NM_170734.4	v6	VIIb / BDNF6B	c	2	1	[2]	263
NM_001143805.1	v7	IIa / BDNF2A	a	0	2	[0,0]	248
NM_001143806.1	v8	IIb / BDNF2B	a	0	2	[0,0]	248
NM_001143807.2	v9	III / BDNF3	a	1	2	[1,0]	248
NM_001143808.2	v10	Va	a	1	2	[1,0]	248
**NM_001143811.2**	**v11**	**V-VIII-VIIIh**	**a**	**11**	**4**	**[1,8,2,0]**	**248**
NM_001143812.2	v12	Vh	a	1	2	[1,0]	248
NM_001143813.2	v13	VIa	a	2	2	[2,0]	248
**NM_001143814.2**	**v14**	**VIb-IXbd**	**a**	**3**	**3**	**[2,1,0]**	**248**
NM_001143816.2	v16	IXabd	a	17	2	[17,0]	248
NM_001143809.2	v17	Vb	d	0	1	[0]	277
NM_001143810.2	v18	V-VIII	e	2	2	[1,1]	330

Table S2

RT-qPCR/RT-ddPCR assays and editing PCR primers used in this study.

All sequences are written 5′ to 3′.

Target	Forward	Reverse
**BDNF_total**	GGATGAGGACCAGAAAGTTCG	GGACATGTTTGCAGCATCTAGG
**BDNF_NM_001709**	GTGTGGACCCCGAGTTCC	CAGCCTTCATGCAACCAAAG
**BDNF_NM_170731**	GGGAGACGAGATTTTAAGACACT	CAGCCTTCATGCAACCAAAG
**BDNF_NM_170732**	CTGGGTTAACTTTGGGAAATGC	CAGCCTTCATGCAACCAAAG
**BDNF_NM_170733**	GCTGCCTTGATGGTTACTTTG	CAGCCTTCATGCAACCAAAG
**BDNF_NM_001143811**	TGCATTCTGACCTATTGACTGG	CAGCCTTCATGCAACCAAAG
**BDNF_NM_001143814**	GGACCCGTGAGGTTTGTG	GGTGGAACTGAGATTAGATGGC
ACTB	CACCATTGGCAATGAGCGGTTC	AGGTCTTTGCGGATGTCCACGT
**RLuc**	GTAACGCTGCCTCCAGCTAC	CCAAGCGGTGAGGTACTTGT
**FLuc**	ACTCTAAGACCGACTACCAGG	GTAGACCCAGAGCTGTTCATG
BDNF editing region	AAGCTCAACCGAAGAGCTAAA	AACTCCAAATCGTCCCTTCTAC

Table S3

Antibodies used in this study.

Target	Host (clone)	Product ID	Manufacturer	Dilution
**Primary Antibodies**				
**anti-BDNF [EPR1292]**	rabbit mAb	ab108319	Abcam	1:5,000
**anti-VCL [hVIN-1]**	mouse mAb	V9131	Sigma-Aldrich	1:5,000
**Secondary Antibodies**				
**anti-rabbit IgG-HRP**	goat	7074	Cell Signalling	1:2,000
**anti-mouse IgG-HRP**	horse	7076	Cell Signalling	1:2,000

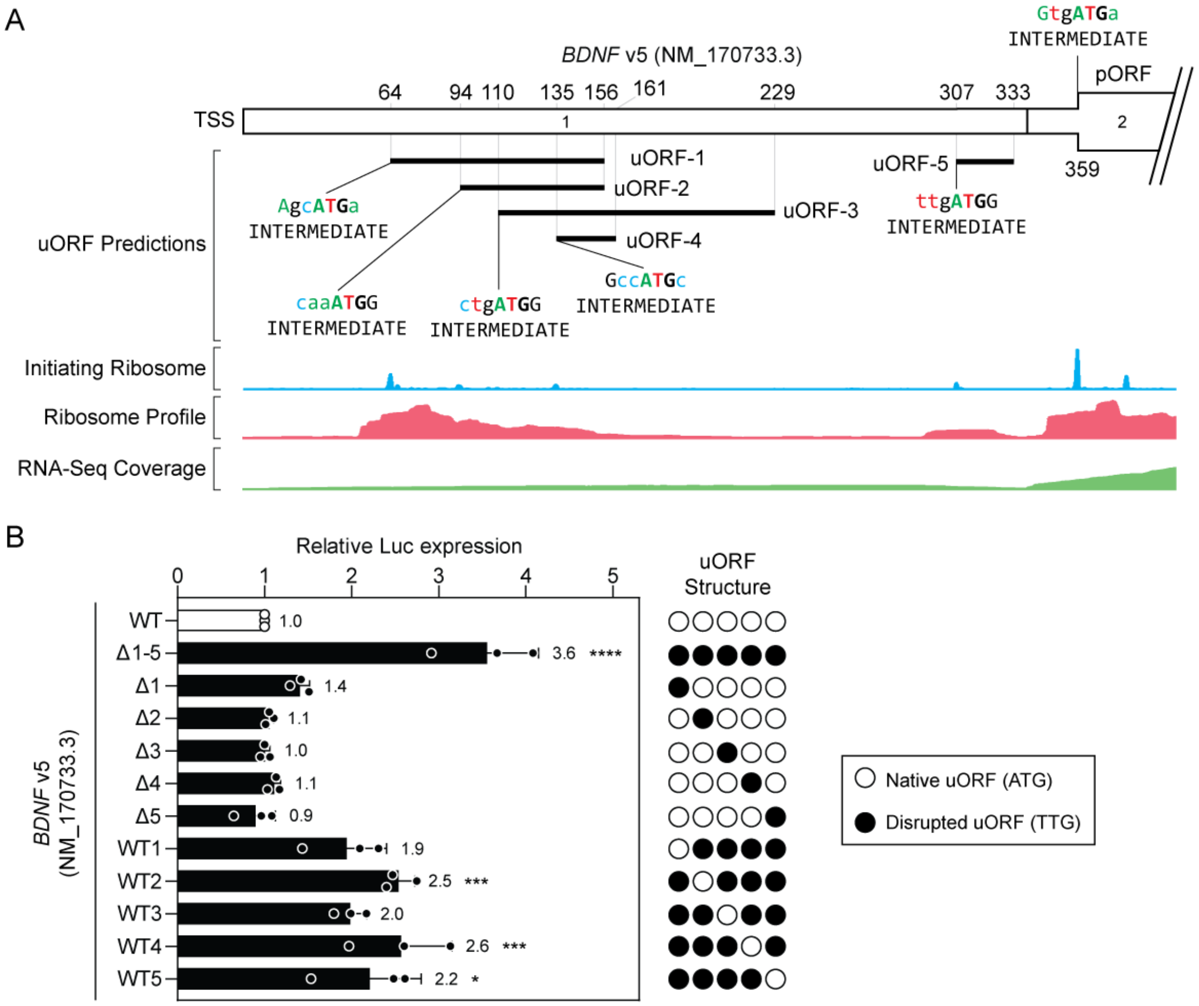

Figure S1

BDNF **transcript v5 is subjected to uORF-mediated repression.**

(**A**) Schematic of *BDNF* transcript v5 NM_170733.4, containing five predicted uORFs. Aggregated Ribo-Seq and RNA-Seq data are overlaid providing evidence of uORF translation. (**B**) HEK293T cells were transfected with *BDNF* v5 5′ UTR dual luciferase reporter constructs as indicated and luciferase activity assayed after 24 hours. uORFs were disrupted by mutation of the corresponding upstream ATG to TTG. Values are mean+SD (n=3 independent experiments), and were scaled such that the mean of the WT control group was returned to a value of 1. Statistical significance was determined by one-way ANOVA with Bonferroni *post hoc* test, *P<0.05, ***P<0.001, ****P<0.0001.

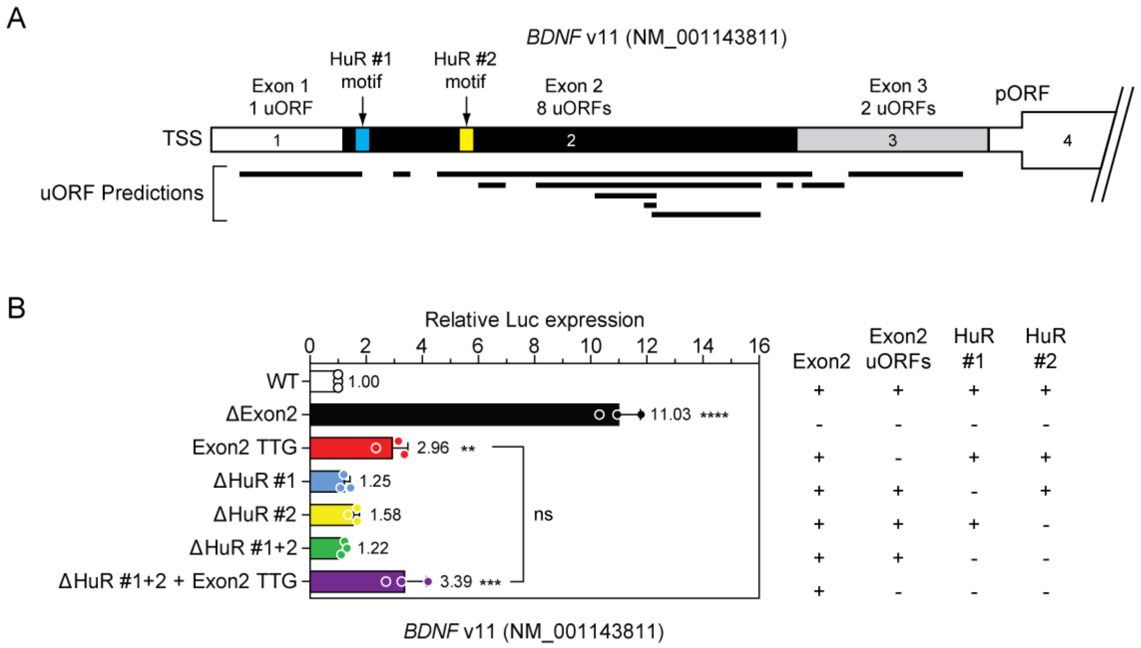

Figure S2

uORFs are only partially responsible for the repressive activity of *BDNF* v11 exon 2.

(**A**) Schematic of the *BDNF* v11 5′ UTR with the HuR #1 and HuR #2 motif sites indicated. The sizes and positions of exons, the locations of predicted uORFs, and the number of uORFs per exon are also indicated. (**B**) HEK293T cells were transfected with various *BDNF* v11 5′ UTR-DLR constructs. Mutants were generated in which either or both of the HuR motifs were deleted. An additional construct in which both motifs were deleted and all exon 2 uORFs were disrupted was tested in parallel. Luciferase activity was determined 24 hours post transfection. Values are mean+SD, n=3 independent experiments. Differences between groups were tested by one-way ANOVA and Bonferroni *post hoc* test. ***P*<0.01, ****P*<0.001, *****P*<0.0001, ns, not significant.

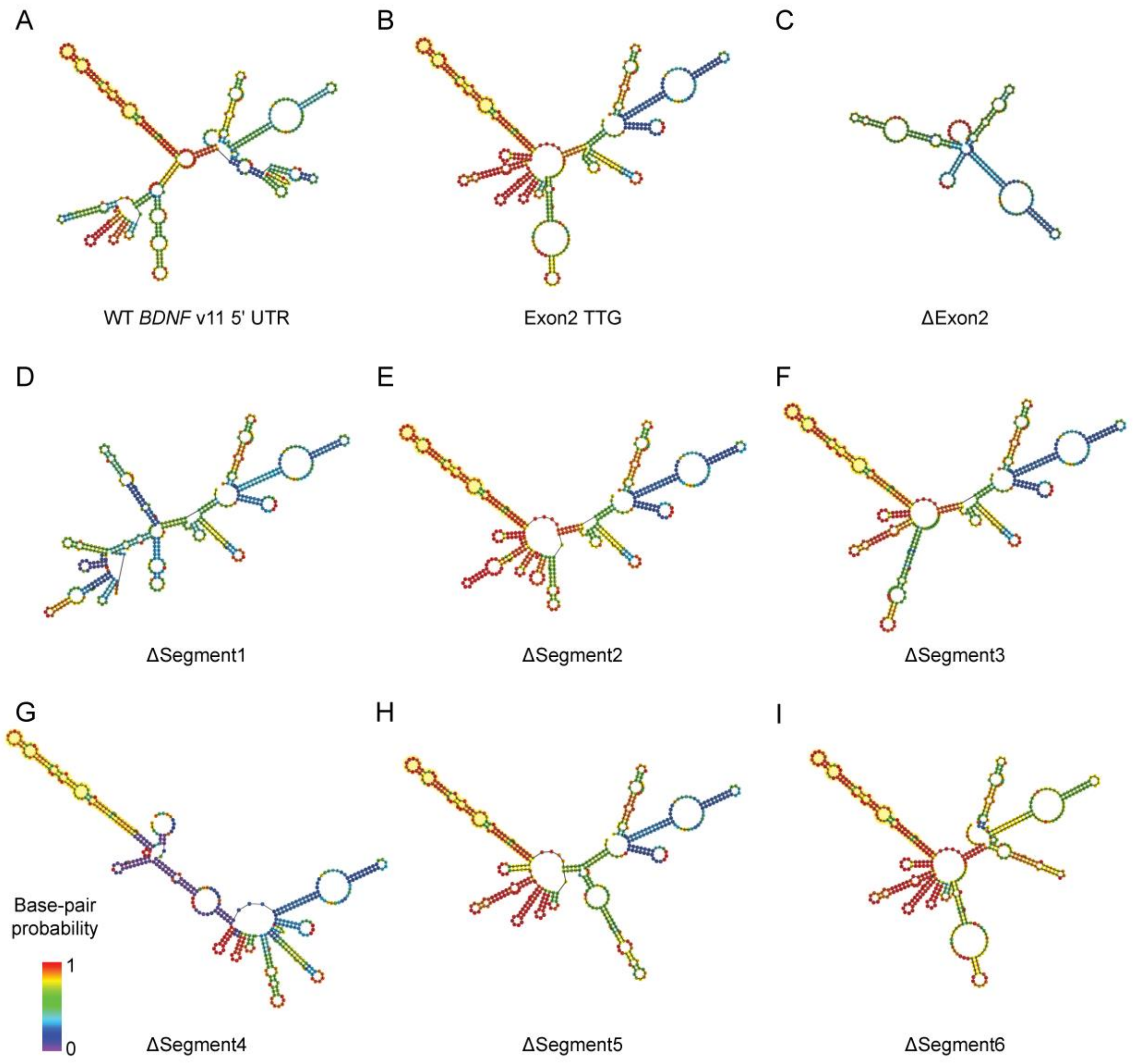

Figure S3

RNA structure predictions for *BDNF* v11 and associated deletion constructs.

RNAfold structures for (**A**) the wild-type *BDNF* v11 UTR, and the *BDNF* v11 5′ UTR whereby; (**B**) all eight exon2 uORFs are disrupted by ATG-to-TTG mutations, (**C**) Exon2 is deleted in its entirety, and (**D-I**) 50 nucleotide segments of exon2 are sequentially deleted (in the context of all exon2 uORFs being disrupted). The hairpin structure of interest is highlighted in yellow.

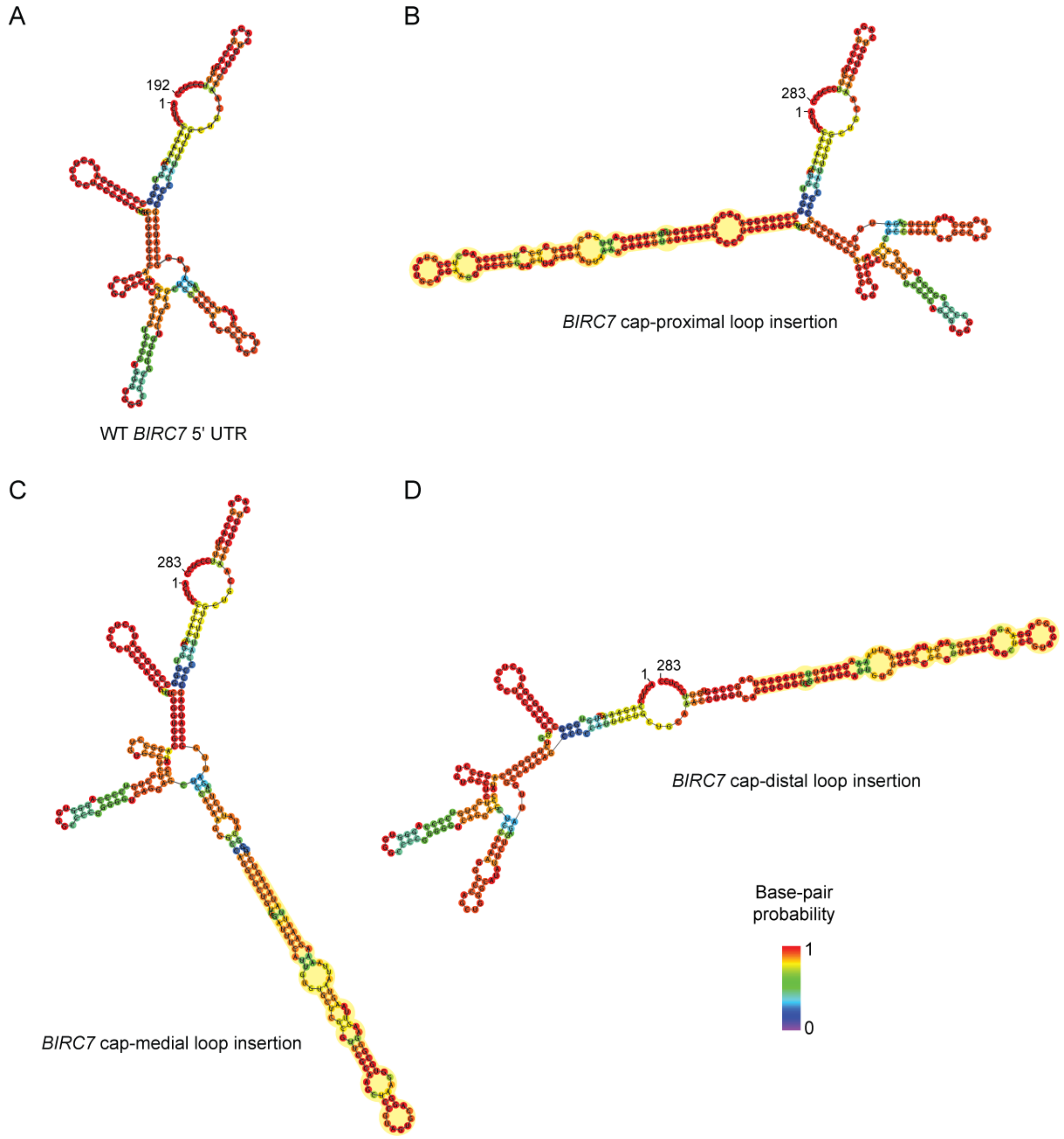

Figure S4

RNA structure predictions for *BIRC7* 5′ UTRs with *BDNF* v11 loop insertions.

RNAfold structures for (**A**) the wild-type *BIRC7* 5′ UTR, and constructs in which a *BDNF* v11-derived loop sequence (highlighted in yellow) is inserted in the (**B**) cap-proximal, (**C**) medial, and (**D**) cap-distal positions.

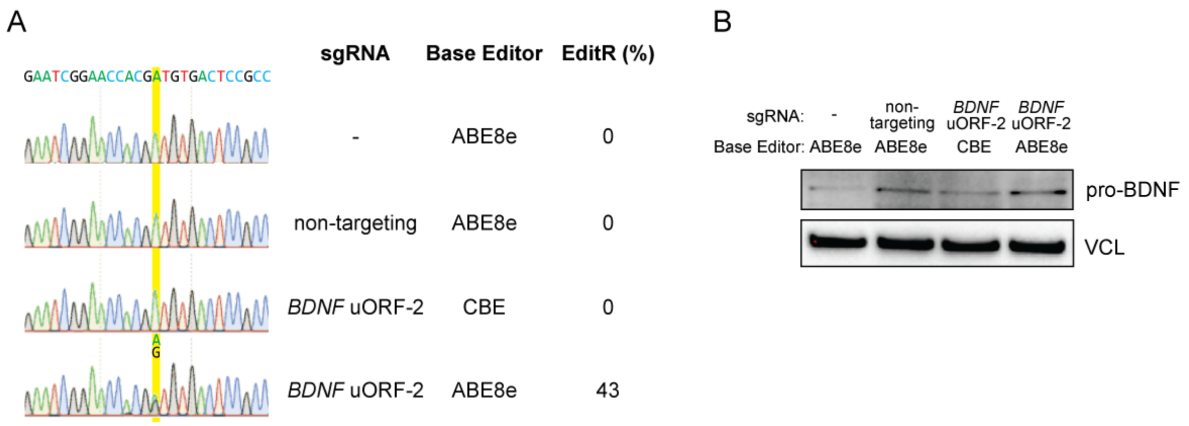

Figure S5

Additional negative controls for *BDNF* v4 uORF-2 base editing-mediated disruption.

HeLa cells were transfected with various plasmids to assess the specificity of adenine base editing of *BDNF* v4 uORF-2. Cells were transfected with the on-target sgRNA or a non-targeting control sgRNA together with an adenine base editor (ABE8e) or a cytosine base edior that is not expected to induce uORF disruption. Editing efficiency was assessed by (**A**) sanger sequencing and EditR quantification analysis, and (**B**) anti-BDNF western blot with Vinculin (VCL) as a loading control.
